# Loose powder detection and surface characterization in selective laser sintering via optical coherence tomography

**DOI:** 10.1098/rspa.2016.0201

**Published:** 2016-07

**Authors:** Guangying Guan, Matthias Hirsch, Wahyudin P. Syam, Richard K. Leach, Zhihong Huang, Adam T. Clare

**Affiliations:** 1 Advanced Manufacturing Technology Research Group, Faculty of Engineering, University of Nottingham, University Park, Nottingham NG7 2RD, UK; 2 School of Engineering, Physics and Mathematics, University of Dundee, Dundee DD1 4HN, UK

**Keywords:** optical coherence tomography, selective laser sintering, additive manufacturing, *in situ* monitoring, part integrity

## Abstract

Defects produced during selective laser sintering (SLS) are difficult to non-destructively detect after build completion without the use of X-ray-based methods. Overcoming this issue by assessing integrity on a layer-by-layer basis has become an area of significant interest for users of SLS apparatus. Optical coherence tomography (OCT) is used in this study to detect surface texture and sub-surface powder, which is un-melted/insufficiently sintered, is known to be a common cause of poor part integrity and would prevent the use of SLS where applications dictate assurance of defect-free parts. To demonstrate the capability of the instrument and associated data-processing algorithms, samples were built with graduated porosities which were embedded in fully dense regions in order to simulate defective regions. Simulated *in situ* measurements were then correlated with the process parameters used to generate variable density regions. Using this method, it is possible to detect loose powder and differentiate between densities of ±5% at a sub-surface depth of approximately 300 μm. In order to demonstrate the value of OCT as a surface-profiling technique, surface texture datasets are compared with focus variation microscopy. Comparable results are achieved after a spatial bandwidth- matching procedure.

## 1. Introduction

Process monitoring and process control methodologies are commonplace for established machining processes, but are not currently applied in additive manufacturing (AM) methods [1]. The high-integrity applications which justify the use of AM require a step-change in the control that users have over the machine technology in order to effect a repeatable and verifiable sintering regime. Defects in selective laser sintering (SLS) have been well catalogued [2]. There has been significant effort to control feedstock, indirectly monitor sintering and control machine processes, which is reported in [3–5] and in Everton *et al*.’s review on the subject [6]. Despite this effort, assurance cannot be provided through current technologies for the success of an operation. Optical coherence tomography (OCT) is proposed here as an excellent method for detecting sub-surface defects in components fabricated by SLS.

OCT has been used in a wide range of applications where the material properties of the object being measured permit sufficient transmission of the incident laser light. There continues to be significant interest in the use of OCT in the biomedical sector for ophthalmic diagnosis [7] and more recently for the identification of near-surface defects in multiple tissue types [8–10]. With respect to applications in manufacturing technology, OCT has also been explored for use in composites manufacture [11,12]. Dunkers *et al*. [13] used OCT to characterize defects that are apparent in the preparation of glass fibre composites, which offered new insight into the material integrity. The data that are captured by OCT when deployed in a manner specific to the process and application are of significant value to manufacturers of high-value parts.

In this study, OCT is used to interrogate the SLS process *ex situ*. In order to classify sub-surface regions of un-melted and partially melted powder, specimens were prepared by varying incident energy density. Intensity profiles are investigated as a tool to measure interface effects and identify simulated failings in the sintering process. OCT has also been compared with a focus variation microscope to assess its capability to measure the surface texture of AM build layers; this demonstrated that OCT is a viable technique for inclusion in future SLS systems where in-build defects cannot be tolerated.

## 2. Material and methods

### (a) Optical coherence tomography arrangement

**Figure 1.**
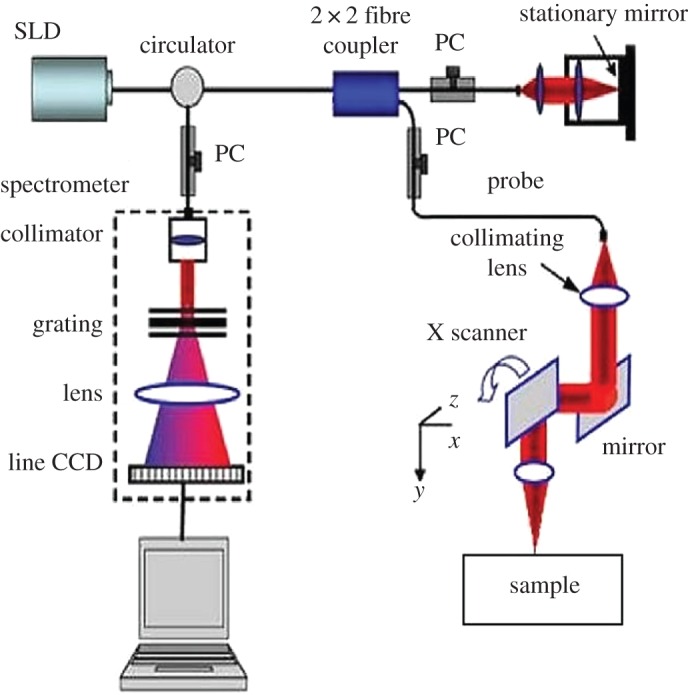
Schematic of the typical spectral domain PhS-OCT system set-up used in this study. (Online version in colour.)

In this work, a spectral domain phase-sensitive OCT (PhS-OCT) system, detailed elsewhere [14], is employed to capture cross-sectional structural images. A simplified schematic diagram of the PhS-OCT system is shown in figure 1. The PhS-OCT system employs a super-luminescent diode (SLD) as the light source, with a peak wavelength of 1310 nm and a bandwidth of approximately 110 nm, implemented in a spectral domain configuration. The sample arm uses an objective lens of 30 mm focal length to deliver the light to the test sample. The OCT system provides an axial resolution of 7.2 μm and a lateral resolution of 15 μm in air. The acquisition rate was determined by the spectrometer employed in the system, which had a maximum rate of 92 000 A-scans (an A-scan is a routine type of diagnostic test which provides data on the length of the eye) per second and generated a frame rate of 180 frames s^−1^ when the A-lines were set to 512 per frame.

A light beam from the SLD is delivered to a fibre-based Michelson interferometer via an optical circulator. The light is then split 10/90 through a fibre coupler, where one arm of the light is directed towards a stationary mirror in the reference arm and the other arm is collimated and directed to the sample via a two-dimensional (2D) scanning galvo-mirror. The reflected light from the reference arm and the backscattered light from the sample arm are sent to a high-speed spectrometer, which is capable of detecting the interference pattern. The spectrometer consists of a 30 mm focal length collimator, a 1200 lines mm^−1^ transmission grating, a 100 mm focal length achromatic lens and a 14-bit, 1024 pixel InGaAs line-scan camera.

**Figure 2.**
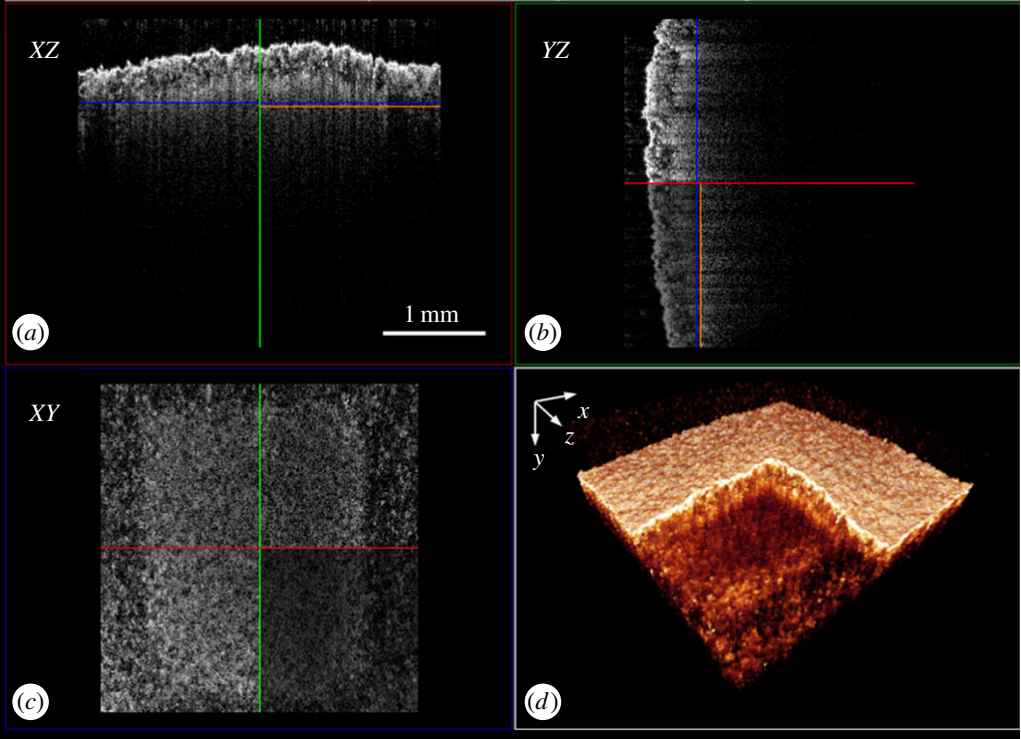
A typical 3D volumetric OCT structural image of a specimen with stark density variation: (*a*) *XZ-*plane view, (*b*) *YZ*-plane view, (*c*) *XY*-plane view and (*d*) 3D view with the top corner cropped. (Online version in colour.)

The method for the acquisition of a cross-sectional 2D structural image in AM specimens is described elsewhere [2]. OCT performs cross-sectional imaging by measuring the magnitude and echo time delay of backscattered light. Cross-sectional images are generated by performing multiple axial measurements of echo time delay (axial scans or A-scans) and scanning the incident optical beam transversely. Images, or B-scans, were generated by multiple A-scans; in this study, one B-scan consists of 512 A-scans. Three-dimensional (3D) volumetric datasets can be achieved by multiple B-scans, which are controlled by the 2D scanning galvo-mirror system. A typical 512 B-scan dataset collection takes less than 3 s. Figure 2*d* shows the 3D structural image of an SLS specimen with the top corner cropped for observation of sub-surface features. Figure 2*a*–*c* shows one B-scan from three planes and an isometric visualization.

### (b) Sample preparation

The test parts analysed in this study were fabricated using a commercially available polyamide-12 laser sintering material (PA2200; EOS GmbH), processed by a FORMIGA P 100 SLS system (EOS GmbH). The specimens were produced with a melting laser scan speed of 2500 mm s^−1^, a hatch spacing of 250 μm and a layer thickness of 100 μm. They were designed to mimic a range of densities of loose powder caused by un-melted/insufficiently sintered volumes in SLS-produced parts. The loose powder areas were designed to reside approximately 100 μm below the surface, the thickness of each layer. The upper ‘skin’ was sintered using standard parameters to create one solid cover layer. The variation in sub-surface powder density was created by modulating the laser power from switch-on to 11 W in equal steps of 1 W.

## 3. Results

### (a) Surface texture measurement by optical coherence tomography and focus variation microscopy

An SLS sample was measured by two different optical instruments: focus variation microscopy (FVM) [15–17] and the OCT system developed here for use with AM specimens. The main goal was to compare the capability of OCT in measuring surface texture (both profile and areal) with that of FVM. For surface texture measurement, the optical instrument is traced to a stylus (contact) instrument. The stylus (contact) instrument measurement result is well understood because mechanical contact between the stylus tip and a measured surface can be well modelled. Owing to this well-understood result, the stylus instrument is used as the reference measurement. Hence, the FVM instrument is verified by comparing it with the stylus instrument. The comparison is carried out by measuring a Rubert 529X reference standard. Comparison data are shown in table 1. These data demonstrate that the results from FVM are in good agreement with the stylus instrument’s results.

**Table 1.** Summary of comparison results for Alicona and Talysurf 50.

profile texture							
description	instrument	vertical resolution (nm)	original sampling distance (μm)	downsampling multiplier (×)	sampling distance (μm)	*L* (evaluation length) (mm)	λs (μm)
Rubert 529X stylus (2 μm tip)	Talysurf 50	16	0.5	1	0.5	1.25	2.5
Rubert 529X (20×)	Alicona	30	0.44	1	0.44	1.25	2.5

description	instrument	λc (mm)	*Ra* (μm)	*Ra* (μm)	*Ra* (μm)	mean (μm)	*σ* (μm)
Rubert 529X stylus (2 μm tip)	Talysurf 50	0.25	0.0826	0.0805	0.0813	0.081	0.001
Rubert 529X (20×)	Alicona	0.25	0.0812	0.0865	0.0866	0.085	0.003

Comparing the profile or areal surface texture data acquired from two different instruments is not a straightforward process and a specific procedure should be followed that accounts for variations in instrument characteristics. Specifically, the measurement and data-processing conditions should be as similar as possible [18].

**Figure 3.**
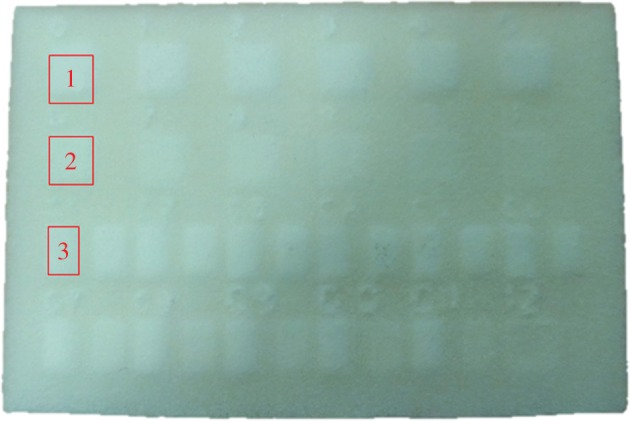
Measurement locations on the SLS sample with sub-surface regions of semi-consolidated powder. (Online version in colour.)

First, the numerical apertures (NAs) of the two instruments should be equal when acquiring the surface data, thereby ensuring that the instruments are measuring the same spatial frequency distribution and have equal slope sensitivities. The FVM measurements were taken by using a 5× objective lens (NA=0.15). The area of measurement of the sample is shown in figure 3, where 1, 2 and 3 correspond to the sample names of SLS 11, SLS 21 and SLS 31, respectively. Both 1 and 3 correspond to the area with sub-surface powders not scanned by the laser or 0 W energy; 2 is the area scanned by 6 W laser energy.

**Figure 4.**
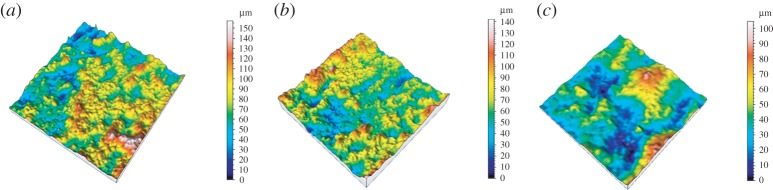
Surface topography data obtained from the OCT instrument. (*a*) SLS 11, (*b*) SLS 21 and (*c*) SLS 31. Note that the area of SLS 31 (*c*) is 400 × 400 μm. (Online version in colour.)

**Figure 5.**
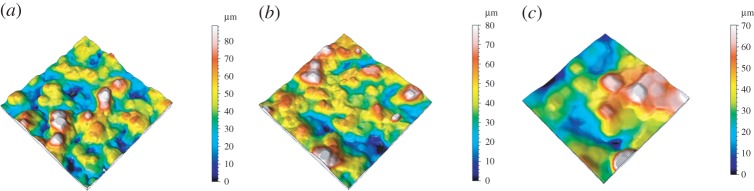
Surface topography data obtained from FVM after resampling. (*a*) SLS 11, (*b*) SLS 21 and (*c*) SLS 31. Note that the area of SLS 31 (*c*) is 400 × 400 μm. (Online version in colour.)

The measurement results from the OCT are in the format of sliced 2D images. For this reason, image processing has to be carried out to extract the profiles from every 2D image in the image stack and combine them into 3D surface data. The sample data were levelled before analysis, by least-squares fitting to a line or plane. The sampling distance (i.e. distance between data points) of the OCT is 6.8 μm, whereas, for FVM, the sampling distance is 1.75 μm. To match the sampling distances, the data from the FVM measurement is numerically resampled, giving a new effective sampling distance of 7 μm. The measurement area for SLS 11 and SLS 21 is 800×800 μm and for SLS 31 it is 400×400 μm. The evaluation lengths *L* for SLS 11 and SLS 21 are 800 μm and for SLS 31 *L* is 400 μm. Figure 4 shows the surface topography data obtained by OCT. Data obtained by FVM after resampling are presented in figure 5.

**Figure 6.**
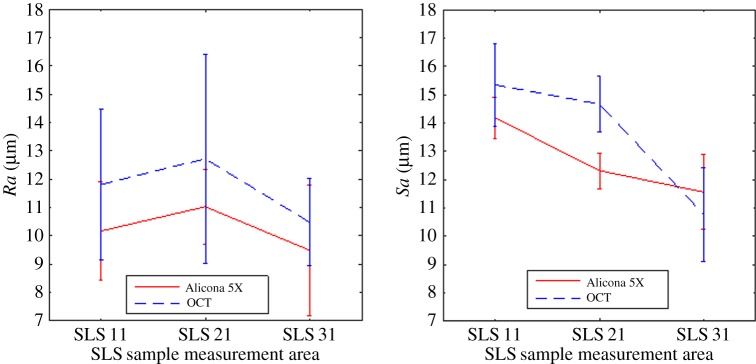
Plot of the calculated *R*a and *S*a from FVM and OCT. The bar indicates the calculated statistical standard deviation (*σ*). (Online version in colour.)

To calculate the profile and areal texture, short-wavelength Gaussian filters [19] (λs of 12 μm for the profile texture and an S-filter with a nesting index of 12 μm for the areal texture) are applied to remove high spatial frequency noise, and to avoid any effects due to the finite sampling distance and optical resolution. Finally, high-pass filtering is applied to the data to ensure matching spatial bandwidths for the two instruments. Owing to the limited evaluation length, only one sampling length was used in the profile analysis (not the default five stipulated in ISO 4287 [20]). Table 2 summarizes the measurement results along with their processing parameters. For each profile and areal texture, five measurements at different positions on the surface were carried out. The mean values and standard deviations were calculated and are displayed in table 2. Plots of the texture results along with their standard deviations are presented in figure 6. From these results, it can be concluded that OCT and FVM can achieve comparable results when measuring the profile and areal texture within the variation across the surface.

**Table 2.** Summary of profile and areal texture with filter parameters.

profile texture							
sample	instrument	original sampling distance (μm)	sampling distance (μm)	*L* (evaluation length) (mm)	λs (μm)	λc (mm)	*Ra* ($\bar{y}\pm 2\sigma$) (μm)
SLS 11	FVM 5×	1.75	7	0.8	12	0.8	10.17 ± 1.74
	OCT	6.8	6.8	0.8	12	0.8	11.81 ± 2.68
SLS 21	FVM 5×	1.75	7	0.8	12	0.8	11.03 ± 1.33
	OCT	6.8	6.8	0.8	12	0.8	12.72 ± 3.7
SLS 31	FVM 5×	1.75	7	0.4	12	0.4	9.49 ± 2.31
	OCT	6.8	6.8	0.4	12	0.4	10.48 ± 1.54

areal texture							
sample	instrument	original sampling distance (μm)	sampling distance (μm)	*L* (evaluation length) (mm)	S-filter (μm)	L-filter (mm)	*Sa* ($\bar{y}\pm \sigma$) (μm)
SLS 11	FVM 5×	1.75	7	0.8	12	0.8	14.18 ± 0.72
	OCT	6.8	6.8	0.8	12	0.8	15.34 ± 1.47
SLS 21	FVM 5×	1.75	7	0.8	12	0.8	12.32 ± 0.63
	OCT	6.8	6.8	0.8	12	0.8	14.67 ± 0.98
SLS 31	FVM 5×	1.75	7	0.4	12	0.4	11.56 ± 1.32
	OCT	6.8	6.8	0.4	12	0.4	10.77 ± 1.67

### (b) Sub-surface powder density measurement

#### (i) Measurements based on intensity density analysis

**Figure 7.**
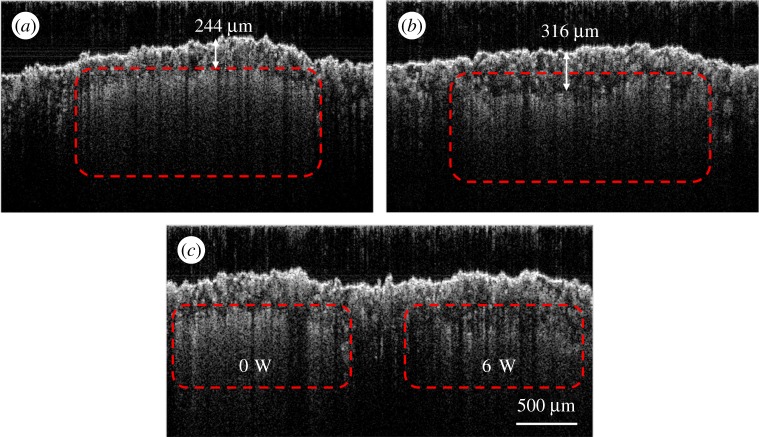
OCT cross-sectional structural imagesof areas with different loose powder density caused by shifting laser energy: (*a*) area with no laser melting, (*b*) area with 6 W, (*c*) no laser interaction and 6 W side-by-side. (Online version in colour.)

The specimens produced were first non-destructively evaluated by OCT and then sectioned for characterization by scanning electron microscopy (SEM). The specimens were designed to have a smooth surface texture and a solid top layer of approximately 100 μm, as described in §2b. However, some surface deformation (bellying) was observed, as shown in figure 7, and the depth of solid varied for each processing area. This is typical for current additive layer methods, where fine feature deformation upon cooling is observed. Furthermore, owing to standard processing parameters used for the top surface, the melting laser power will consolidate more than the currently added material of the layer. In solid, this would be considered re-melting. Precise control of the laser power to only melt a single layer is not given by the commercial manufacturing process. Figure 7*a*–*c* shows the OCT cross-sectional structural images at the central sections of SLS 11, SLS 21 and SLS 31, respectively, which are marked by 1, 2 and 3 as in figure 3. Figure 7*a* shows the controlled area, which was not subject to laser melting, with a solid top layer of approximately 244 μm; Figure 7*b* shows an area treated with 6 W laser power with a top layer of approximately 316 μm; and Figure 7*c* is the area containing both treatments with an uneven depth of the top layers in one frame. The suspected region of un-melted/insufficiently sintered area, which is filled with loose powder, is indicated by the dashed line.

**Figure 8.**
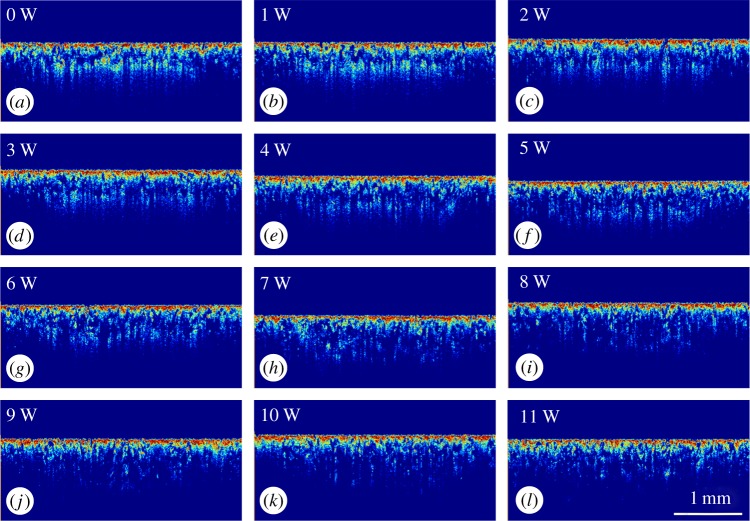
Panels (*a*–*l*) are the normalized OCT cross-sectional structural images of areas ramping up from no laser interaction to 11 W. (Online version in colour.)

**Figure 9.**
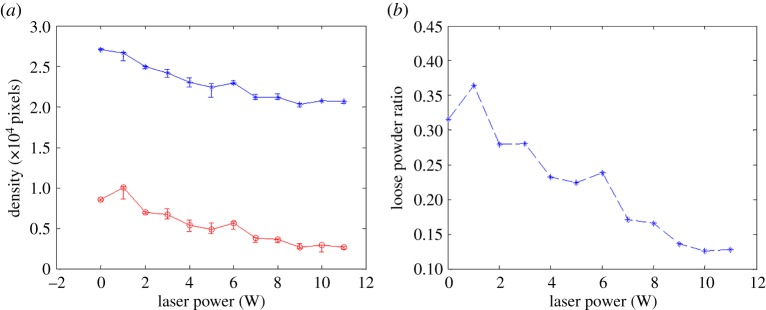
Relationship between (*a*) laser energy and (*b*) loose powder density; standard deviation was calculated from five datasets. (Online version in colour.)

Figure 8*a*–*l* shows the OCT cross-sectional structural images of areas corresponding to laser power from no laser interaction to 11 W. The images were normalized and converted from greyscale images to scaled colour images for better contrast between the solid and loose powder areas. Each structural image was pre-processed to have a flat surface for the convenience of using a square window to capture the loose powder area for quantitative density analysis. The OCT signal is derived from the backscattering of light from the sample, hence the higher intensity is a result of higher backscattering properties, which show as a brighter colour in figure 8*a*–*l*. Pixel numbers were used, where intensity is above a predetermined threshold, to calculate sample density. It can be observed from figure 8*a*–*l* that the apparent sub-surface loose powder density is reduced gradually when the laser energy is increased. This is to be expected as the degree of sintering is significantly affected by the power delivered to the powder bed. Quantitative analysis of the density change is shown in figure 9. The blue curve in figure 9*a* shows the total pixel number, as the density of the whole sample, from each structural image in figure 8 and the red curve shows the pixel numbers of loose powder areas, as the density of loose powder. It can be observed from figure 9*a* that both loose powder density and whole sample density reduced gradually as the laser power was increased. The relationship between the laser power and the averaged density ratio of the loose powder and the whole sample density is shown in figure 9*b*. This method can easily distinguish the sample density change when the laser power increases to 9 W; however, the density or density ratio change is difficult to detect once the power is greater than 9 W. The OCT signal pixel numbers reduced when the laser power was increased, which is due to the increase in the sufficiently sintered area, less backscattering and hence reduced light penetration depth.

**Figure 10.**
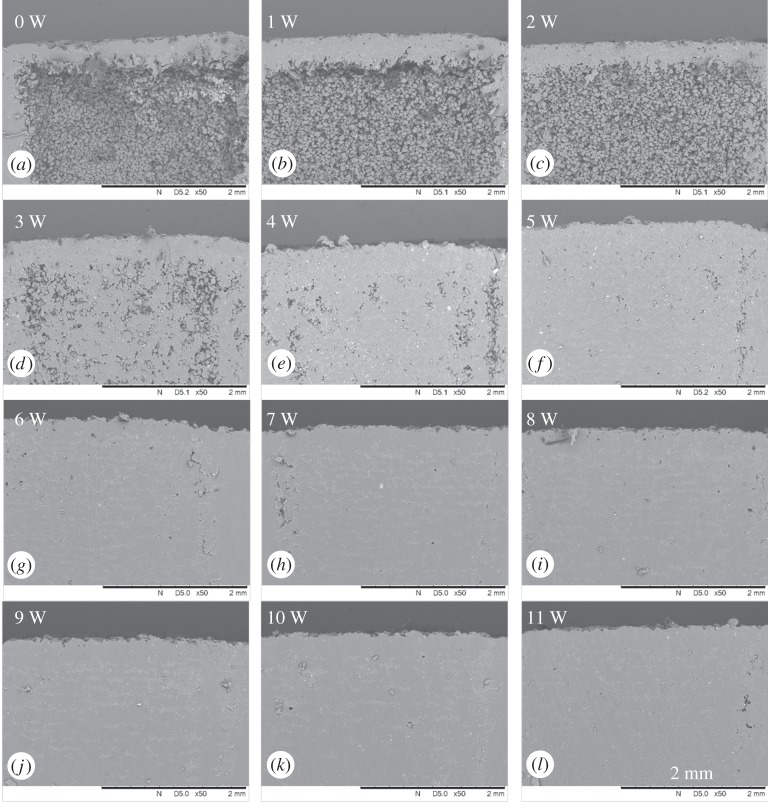
Panels (*a*–*l*) are SEM images of areas corresponding to laser power from no laser interaction to 11 W.

Figure 10 shows SEM images of the areas corresponding to laser powers from 0 W to 11 W. Loose powder is liberated in figure 10*a* after cutting, because there was insufficient incident power to cause even mild sintering. Significant density and melt status changes can be observed from 0 W to 5 W, which are shown in figure 10*a*–*f*, respectively. However, it is difficult to distinguish a difference when the incident laser power exceeded 6 W, which indicates that OCT may be a better analysis tool for post-build investigations than cross sectioning. It is probably in the preparation of sections that some porosity is obscured by smearing of the polished surface. This is a common problem that occurs in specimen preparation of AM cross-sections.

#### (ii) Measurements based on intensity attenuation analysis

It can be observed from figures 7 and 8 that the intensity attenuation coefficient throughout the depth for the solid and loose powder areas varies significantly as a result of the variation in processing parameters. This is due to more specular reflection from the melted area when compared with the diffuse reflection from the loose powder area. This difference in reflection type and the detection of loose powder with open surfaces have been reported elsewhere [2]. In this study, the focus was on sub-surface loose powder detection and image reconstruction for automatic defect detection by OCT.

**Figure 11.**
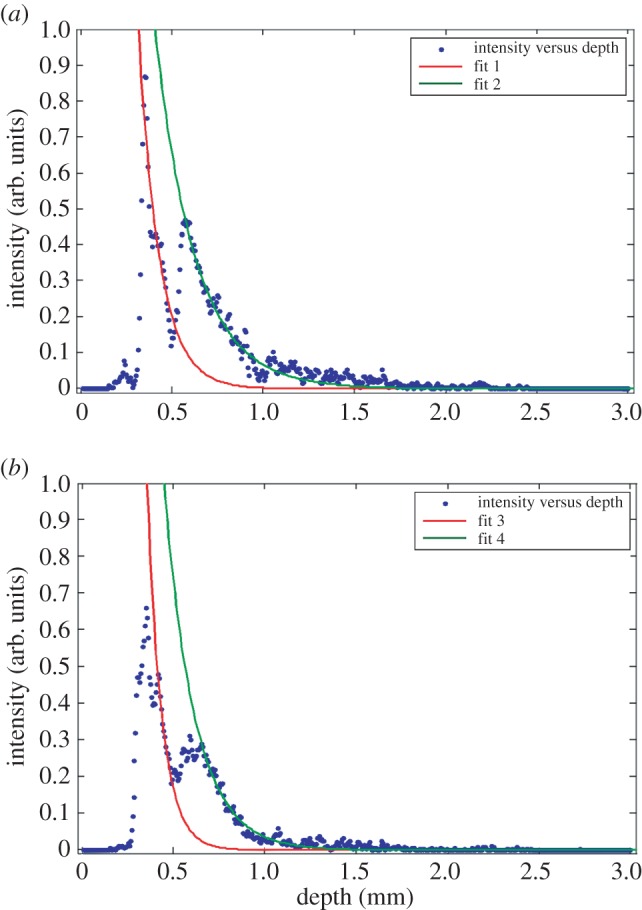
Panels (*a*) and (*b*) are the averaged intensity change throughout the depth from the 0 W and 6 W areas, respectively. The attenuation coefficients for fit 1 to fit 4 are −8.821, −4.565, −12.4 and −6.027, respectively. (Online version in colour.)

The results of the intensity attenuation coefficient analysis are shown in figure 11. Figure 11*a*,*b* shows depth-resolved intensity changes from selected areas from 0 W and 6 W specimens, respectively. In this study, one OCT B-frame image consists of 512 A-lines with an equal spacing of 6.8 μm. A mean of 10 adjacent A-lines from each section has been calculated in order to enhance the signal-to-noise ratio.

Quantitative analysis was performed by applying an exponential curve fit to the intensity profile of each selected area. The highest peak from each section represents the location of the surface. The consecutive 15 pixels below the surface were not used in the fitting process in order to eliminate the high reflection from the air–sample interface. The cut-off intensity threshold was set to 15% of the peak value to avoid the artefact signal originating from deeper within the sample. The exponential fitting coefficients for fit 1 and fit 2 in figure 11*a* are −8.821 and −4.565, respectively. If the intensity drop-off was purely due to absorption in polyamide, then the ratio of the two fitting coefficients implies a loose powder attenuation of 52% compared with that of the solid part. The fitting coefficient values for the fits in figure 11*b* are −12.4 and −6.027, respectively, which generates a ratio of 48%. The absolute value of the intensity attenuation coefficient is highly intensity dependent and not suitable for comparison between different image frames or areas. However, the ratio of the attenuation coefficient between the loose powder and the solid area is relatively stable, which could be used to differentiate between these two types of area in an industrial machine tool.

An algorithm for automatically detecting sub-surface loose powder area was also developed based on the intensity attenuation coefficient. This method is similar to previous studies of optical coherence elastography [14,21]. Results from figure 11 state that the absolute attenuation coefficient value at full melt area is higher than that at the un-melted/insufficiently sintered area, however this value is not sufficient for comparison between different B frames (sample areas).

**Figure 12.**
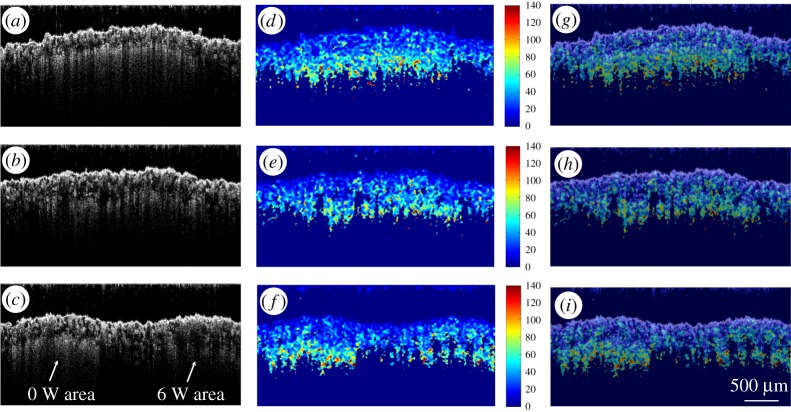
Panels (*a*–*c*) are the structural images of the sample area treated with 0 W, 6 W and both energy values in one B frame, respectively. Panels (*d*–*f*) are the calculated loose powder mappings for the three samples and the colour bars are shown in arb. units; panels (*g*–*i*) are overlaid images. (Online version in colour.)

Each A-line consisted of 512 pixels. Given that in a real specimen in a typical use case the user would not have *a priori* information about the location of detects, a small window of 10 pixels was selected and it was assumed that the intensity attenuation is constant within that range. As the average size of a PA2200 particle used here is approximately 50 μm, this window is large enough to minimize the sensitivity to noise but small enough to return sufficiently high resolution. The reconstruction algorithm consisted of fitting an exponential equation to 10 consecutive pixels, and determining the attenuation coefficients of the exponential fit. Therefore, the attenuation coefficients of pixels 1–10 were obtained, and recorded at pixel 1; then the slope of pixels 2–11 were obtained and recorded in pixel 2; and so on. We were able to ignore the last 10 pixels, as they belong to deep structures where there is almost no signal. To highlight the defect areas, the reciprocals of the attenuation coefficients were used and scaled. Finally, a median filter was used on the reconstructed functional image to minimize noise. The reconstruction results are shown in figure 12. These clearly show the skin region and un-melted powder region below. This is most useful information when evaluating the integrity of the near surface of SLS components where fusion between layers in a process is a key concern.

## 4. Discussion

Surface texture measurements were carried out by means of OCT and FVM, as described in §3a, and comparable results were obtained. The advantage of OCT is that it can evaluate the sub-surface structure alongside evaluating the upper surface similar to other non-contact (optical) instruments. For FVM, as the objective lens used is 5×, which has a low NA, it is difficult to measure a much higher slope surface. To increase the level of confidence in the surface texture measurement results obtained by FVM, they have been compared with the results obtained by a stylus (tactile) instrument (reference instrument).

In §3b, two methods were used to detect sub-surface loose powder areas. First, the OCT signal intensity density was used to evaluate the specimen density. Once PA2200 particles are fully melted and sintered together, the specular reflection increases while the backscattering decreases, which reduces the OCT signal points and penetration depth. Using the pixel numbers with a positive intensity value as a sample density, a linear relationship can be observed between the laser power and the loose powder density. The sensitivity of this method is limited above an incident laser power of over 9 W. However, the performance of this method is still superior to SEM evaluations of cross-sections. The other method makes use of intensity attenuation-level analysis. Previous work has proven that the OCT intensity attenuation coefficient is distinct for the solid and exposed loose powder area and the ratio of the coefficient is quite constant [2]. In this study, a similar phenomenon is found for sub-surface loose powder areas. The reconstruction algorithm is developed for automatic detection of loose powder defects. The algorithm is based on exponential fitting of a selected window of pixel numbers, which limited the resolution of the functional image.

OCT has showed great potential as a non-destructive evaluation method for *in situ* SLS process monitoring. The main challenge is the scanning speed and area size. The typical working area for an SLS machine is 25 × 25 cm, while a typical OCT scanning area ranges from 3 to 5 mm, controlled here by a galvo-mirror. In this study, the maximum capturing line rate of OCT is 92 kHz, which can achieve 180 frames s^−1^ when the A-lines are set to 512 per frame at a scanning distance of 3.5 mm. For real-time monitoring of the SLS printing process for a large sample, this speed will not be sufficient currently. However, 500 kHz OCT systems [22] are now available, offering a 5.4 times faster scan rate than the current system. In addition, selective sampling could be undertaken to reduce the scanning area in order to match the ‘write’ speed of the SLS system. The rapid development of optical sensor and computing technology makes real-time monitoring and control through dynamic feedback a distinct prospect. One solution is to combine OCT with other detectors, such as a high-speed camera to perform the rough scan. Once the area of interest is located, the OCT can apply a higher resolution scan and provide detailed evaluation of the sub-surface.

One of the most attractive attributes of OCT is its ability to provide depth-resolved cross-sectional images. This means that it has the capability of providing layer-by-layer information for use in defects analysis. Should penetration depths also be increased this will serve to further reduce the time penalty for using OCT instrumentation in SLS systems.

## 5. Conclusion

This study has demonstrated the feasibility of using OCT for surface texture measurement and sub-surface loose powder detection of polymeric parts manufactured using SLS. Experiments reported here indicate that:

— the results of the surface texture measurement, after the bandwidth matching procedure, show that the surface texture measurement results obtained by OCT are comparable with those obtained by FVM;
— from the results of surface and sub-surface loose powder detection, OCT shows a promising capability to distinguish loose powder defects in a solid specimen. Also, this is the first time that an automatic loose powder defects detection algorithm has been developed based on OCT technology; and
— this technique presents itself well as a potential method for *in situ* analysis of SLS parts for ensuring part integrity and verifying process stability.

## Data accessibility

All data are contained within the paper.
